# Antipsychotic prescription patterns among schizophrenia patients in Guangdong Province, China’s 686 program: A retrospective study

**DOI:** 10.1097/MD.0000000000039629

**Published:** 2024-09-13

**Authors:** Yingji Hu, Jinghua Su, Xuanyu Xu, Junyu Li, Shujia Zhang, Xiaodong Chen

**Affiliations:** a Guangzhou Medical University, Guangzhou, China; b Department of Chronic Psychiatry, The Affiliated Brain Hospital, Guangzhou Medical University, Guangzhou, China; c Key Laboratory of Neurogenetics and Channelopathies of Guangdong Province and the Ministry of Education of China, Guangzhou Medical University, Guangzhou, China; d Department of Social Psychiatry, The Affiliated Brain Hospital, Guangzhou Medical University, Guangzhou, China.

**Keywords:** 686 Program, Antipsychotic medications, Polypharmacy, Schizophrenia

## Abstract

Schizophrenia is a severe mental disorder. However, there is limited data on the prescribing patterns of patients under China’s Central Government Support for the Local Management and Treatment of Severe Mental Illnesses Program, known as the “686 program.” This study aimed to investigate the use of antipsychotic medications and associated factors among discharged schizophrenia patients in Guangdong Province, within the 686 Program. This study encompassed schizophrenia patients who were discharged from the Affiliated Brain Hospital, Guangzhou Medical University and enrolled in the 686 Program between January 2019 and December 2019. A total of 1645 hospitalized schizophrenia patients were included in the analysis. Clinical and sociodemographic data were acquired from medical records upon discharge. A total of 15 unique antipsychotic medications were utilized, comprising 4 first-generation (FGAs) and 11 second-generation (SGAs) options. FGAs were prescribed at a rate of 8.3%, while SGAs dominated at 98.8%. Risperidone (40.8%), olanzapine (30.2%), clozapine (24.6%), and amisulpride (15.4%) emerged as the top 4 prescribed medications. Additionally, mood stabilizers were used by 20.4%, antidepressants by 14.8%, sedative-hypnotics by 33.6%, anticholinergics by 26.9%, and other internal medicine drugs by 46.4%. Notably, 60.5% received antipsychotic monotherapy (AMT), while 39.5% underwent antipsychotic polypharmacy (APP). Predictors of polypharmacy included multiple hospital admissions, longer hospital stays, and undergoing modified Electroconvulsive Therapy (mECT) during hospitalization. In Guangdong Province, China’s 686 Program, hospitalized patients commonly receive multiple antipsychotic medications simultaneously. Due to the varying outcomes in current studies on the benefits and risks of polypharmacy, it’s vital to educate psychiatrists about the importance of AMT to reduce APP. Additionally, randomized, controlled trials are essential to identify the safest and most effective antipsychotic combinations, as well as to understand which patient profiles may benefit from these combinations.

## 1. Introduction

Schizophrenia comprises a group of severe mental conditions of unknown etiology. It is characterized by a high level of comorbid mental disorders, premature mortality, and substantial socioeconomic burdens.^[1–3]^ Approximately 24 million individuals worldwide are affected by schizophrenia, equating to 1 in every 300 people.^[4]^ According to a systematic review report on schizophrenia in China, the prevalence rate of schizophrenia is 0.5% in rural areas and 0.83% in urban areas. This accounts for a total of 7.16 million individuals affected during their lifetime.^[5]^

Currently, antipsychotic pharmacotherapy remains the cornerstone in treating schizophrenia. This encompasses both first-generation antipsychotics (FGAs) like chlorpromazine, haloperidol, and sulpiride, and second-generation antipsychotics (SGAs) such as clozapine, risperidone, and olanzapine. Emerging perspectives also include third-generation antipsychotics like aripiprazole and cariprazine.^[6,7]^ Variability in efficacy, safety, and side effects, including metabolic syndrome, constipation, cardiovascular complications, prolactin elevation, and extrapyramidal symptoms, characterizes these medications.^[8–10]^ Prescription patterns of antipsychotic medications in various regions and countries are influenced by both clinical and non-clinical factors. These factors encompass the pharmacological expertise of psychiatrists, medication expenses, healthcare policies, insurance coverage, patient age, and psychiatrist preferences. As a result, there is significant variability in the choices of clinicians related to antipsychotic drugs.^[11–15]^ Despite guidelines advocating for antipsychotic monotherapy (AMT),^[16]^ approximately 20% of patients exhibit inadequate response or resistance, prompting consideration of antipsychotic polypharmacy (APP).^[17,18]^ While controversial, APP’s potential benefits in efficacy enhancement and adverse reaction mitigation, exemplified by aripiprazole’s impact on prolactin levels, are noted.^[19]^ However, concerns regarding drug interactions persist, with varying prescription patterns observed globally.^[20]^ For instance, at Prof Dr Alexandru Obregia’Clinical Psychiatry Hospital in Romania, 64.25% of 193 schizophrenia patients hospitalized in 2019 received AMT, while 35.75% were given APP, with the most common combination being paliperidone and clozapine (14.5%). Furthermore, clozapine and risperidone (13%) were also frequently prescribed.^[21]^ In China, a 2019 study involving 1032 patients with schizophrenia found 56.9% received AMT, primarily olanzapine (29.5%). Meanwhile, 43.1% were prescribed APP, with the top combinations being clozapine + risperidone (17.5%) and olanzapine + risperidone (9.9%).^[22]^ Therefore, a significant difference can be observed in the choice of antipsychotic medications between countries. Studies predominantly focus on sociodemographic factors, necessitating a deeper exploration of patient-related variables influencing APP. Amidst this, clinicians advocate for rational psychopharmacotherapy to optimize treatment outcomes while minimizing adverse effects on patient quality of life. Consequently, an in-depth analysis of prescription trends among schizophrenia patients is imperative.

Mental health challenges, a global concern including in China, prompted the inception of the “Central Government Support for the Management and Treatment of Severe Mental Disorders” program in December 2004. After the initial fiscal allocation of 6.86 million yuan, it was named as 686 Program.^[23]^ The long-term objective of this program is to establish a community-based, function-oriented, multidisciplinary, and patient-centered mental health service system for those suffering from severe psychiatric conditions. By 2020, this initiative, implemented nationwide, served 6.43 million patients, with schizophrenia patients comprising the majority.^[24,25]^ Praised for establishing a comprehensive community mental healthcare system, the 686 Program aims to integrate treatment, enhance treatment rates, and raise community awareness about severe mental illnesses.^[26]^ By establishing a national database, it offers a resource for policy formulation and treatment strategies. However, the prescription patterns of schizophrenia patients within the 686 Program remain understudied. Exploring these patterns provides invaluable insights for community psychiatrists, enabling more comprehensive and continuous care, potentially mitigating social disconnection issues from prolonged hospitalization, and facilitating patients’ reintegration into community life. The objective of this study is to fill some of the gaps in local research.

This research investigated the antipsychotic prescription patterns of schizophrenia patients discharged from the Affiliated Brain Hospital, Guangzhou Medical University and enrolled in the 686 Program. A broad range of sociodemographic and clinical characteristics potentially related to APP were investigated. This enabled an assessment of whether current local practices align with standard treatment guidelines and provided a deeper understanding of the complexity of APP practices within the 686 Program.

## 2. Methods

### 2.1. Experimental design and study population

This study is retrospective and utilized clinical records of patients treated at the Affiliated Brain Hospital, Guangzhou Medical University. Participants eligible for the study were those discharged between January 2019 and December 2019 with a diagnosis of schizophrenia according to ICD-10 and enrolled in the 686 Program. If a patient had multiple admissions (≥2) within 2019, the clinical records from the first admission were used.

The study was conducted in accordance with the latest version of the Declaration of Helsinki. Moreover, it was approved by the Ethics Committee of the Guangzhou Medical University Affiliated Brain Hospital (2020-048). Informed consent was not required for this trial, as data were extracted from the Hospital Information Management System and patient data were anonymized.

### 2.2. Procedures

Sociodemographic, clinical characteristics, and medication details at discharge of eligible patients were retrospectively retrieved from the hospital information system. This information was gathered and recorded by psychiatrists using an electronic questionnaire.

### 2.3. Measures

Demographic features comprised age, education level (illiterate, primary or secondary school, university or above), marital status (unmarried, married, divorced/separated/widowed), and payment method (employee health insurance, resident health insurance, fully self-paid, other).

Clinical features encompassed the number of schizophrenia episodes (1, 2–4, ≥5), the number of hospitalizations due to schizophrenia (1, 2–4, ≥5), length of hospital stay, mode of admission (voluntary, involuntary), insight (absent, partial, full), presence of comorbid physical illness, history of risky behaviors, and history of receiving modified Electroconvulsive Therapy (mECT).

To assess the severity of general psychopathological symptoms, the Clinical Global Impression-Severity Index (CGI-SI) rating was utilized. The CGI-SI, recognized for its high reliability and validity, serves as an effective and reliable tool for the assessment of mental illnesses. Moreover, it is widely used among patients with mental disorders.^[27]^ The assessments conducted using this tool mainly include mental state, disease symptoms, daily living abilities, and treatment effects. The severity of general psychopathological symptoms is scored on a 4-point scale, with 4 denoting “severe, significantly troubled by symptoms,” 3 indicating “moderate, somewhat influenced,” 2 representing “mild, hardly influenced,” and 1 signifying “no symptoms or questionable symptoms.” The CGI-SI score shall be assessed by the attending physician upon patient admission.

At discharge, medication details encompassed the prescription of antipsychotic drugs, mood stabilizers, antidepressants, sedative-hypnotics, anticholinergic drugs, and other internal medicine drugs (such as antihypertensives, lipid-lowering drugs, anti-diabetic drugs, gastroprotective drugs, traditional Chinese medicine, etc.).

### 2.4. Statistical analyses

SPSS version 25.0 (IBM SPSS 25.0, Chicago) was utilized to perform statistical analyses, with significance levels defined as *P* < .05. To mitigate the risk of Type I errors resulting from conducting multiple statistical tests, we implemented the Bonferroni correction method. Initially, the prescription patterns of schizophrenia patients were summarized, focusing on the specificity of antipsychotic drugs and their usage rates, the use of anticholinergic drugs, the usage rates of other types of psychiatric drugs, and the use of other internal medicine drugs. There is controversy in schizophrenia regarding the use of AMT versus APP. Therefore, this study focused on investigating single versus multiple antipsychotic drug prescriptions. For the data, continuous variables were subjected to the t-test, whereas categorical variables underwent analysis using the chi-square test. The univariate and multivariate logistic regression models were employed for comparisons of clinical variables between the AMT and APP groups. All variables were included as independent variables.

## 3. Results

### 3.1. Prescription patterns among patients of schizophrenia

**Figure 1.**
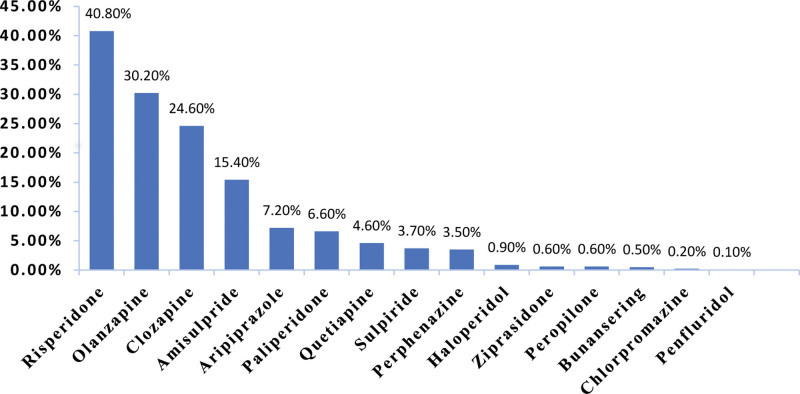
Analysis of antipsychotic medication use among schizophrenia patients (N = 1645).

The study included a total of 1686 patients diagnosed with schizophrenia according to ICD-10 and enrolled in the 686 Program from January 2019 to December 2019. Out of these, 41 patients had incomplete clinical medication information or sociodemographic data, resulting in 1645 patients (97.6%) eligible for analysis. There were 786 females (47.8%) with an average age of 38.04 ± 15.54 years, and the average level of education was secondary school, with an average illness duration of 11 months. A total of fifteen antipsychotic medications were prescribed, consisting of 11 SGAs and 4 FGAs (Fig. 1). The 4 most commonly prescribed antipsychotic medications, based on usage rate, were: risperidone (40.8%, 671/1645), olanzapine (30.2%, 496/1645), clozapine (24.6%, 405/1645), and amisulpride (15.4%, 254/1645).

The usage rates for FGAs and SGAs were 8.3% (137/1645) and 98.8% (1625/1645), respectively. Out of the total patients, 60.5% (995/1645) received treatment with a single antipsychotic medication, while 39.5% (650/1645) were prescribed a combination of multiple antipsychotic medications.

Mood stabilizers were utilized by 20.4% (335/1645) of the patients, with valproate (81.5%, 273/335) and lithium carbonate (15.5%, 52/335) being the most commonly used. Antidepressants were prescribed to 14.8% (243/1645) of the patients, with sertraline (25.1%, 61/243), escitalopram (14.0%, 34/243), and venlafaxine (12.3%, 30/243) being the most frequently utilized. Benzodiazepines and non-benzodiazepine sedative-hypnotics were used by 33.6% (553/1645) of the patients, with lorazepam (36.9%, 204/553), oxazepam (18.8%, 104/553), and alprazolam (13.9%, 77/553) being the most used. Anticholinergic drugs were prescribed to 26.9% (443/1645) of the patients. Other internal medicine drugs were used by 46.4% (764/1645) of the patients, with antihypertensive (32.6%, 249/764) and antidiabetic medications (19.1%, 146/764) being the most common.

### 3.2. Prescription patterns for antipsychotic monotherapy and polypharmacy

**Figure 2.**
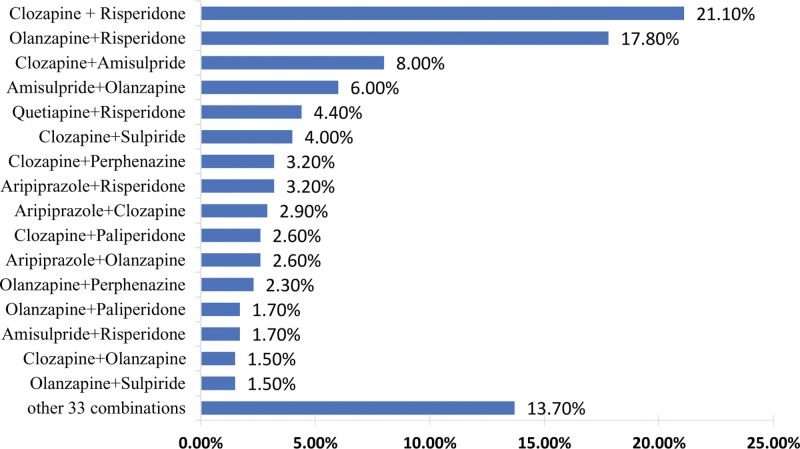
Prescription patterns of combined antipsychotic polypharmacy in schizophrenia patients (N = 650). *89 cases of other 33 combined antipsychotic medication prescription patterns: includes 86 cases with 2-drug combinations and 3 cases with 3-drug combinations. Each combination’s proportion is less than 1.3%.

Among the 650 patients receiving APP treatment, 99.5% (647/650) were prescribed 2 antipsychotic medications, and 0.5% (3/650) were prescribed 3 antipsychotic medications. Figure 2 displays the common patterns of APP. The 3 most common APP combinations were clozapine + risperidone (21.1%, 137/650), olanzapine + risperidone (17.8%, 116/650), and clozapine + amisulpride (6.0%, 39/650). The top 4 antipsychotic medications prescribed to patients receiving AMT were: risperidone (34.8%, 346/995), olanzapine (26.8%, 267/995), amisulpride (13.6%, 135/995), and clozapine (11.1%, 110/995).

### 3.3. Comparison of clinical features between antipsychotic monotherapy and polypharmacy

Table 1 presents the clinical features of both groups. Statistically significant variances were observed in the total number of episodes, frequency of hospitalization, length of hospital stay, and utilization of mECT among patients treated with AMT versus APP (*P* < .05). Logistic regression was performed to adjust for covariates (Table 2). Patients with a history of multiple hospitalizations (odds ratio [OR], 1.786, 95% confidence interval [CI] [1.253–2.545]), longer hospital stays (OR, 1.003, 95% CI [1.002–1.005]), or who underwent mECT during hospitalization (OR, 1.922, 95% CI [1.502–2.459]) were more likely to receive APP treatment compared to the AMT group.

**Table 1:** Comparison of characteristics between schizophrenia patients on antipsychotic monotherapy (AMT) and those on combined antipsychotic polypharmacy (APP) (N = 1645).

Variables	Monotherapy (n = 995)	Polypharmacy (n = 650)	Total (n = 1645)	Chi^2^/Z	*P* value
Socio-demographic characteristics					
Age (years ± SD)*	38.47 ± 15.89	37.39 ± 14.97	38.04 ± 15.54	1.379	.168
Marital status				7.650	.022
Unmarried	545 (54.8%)	392 (60.3%)	937 (57.0%)		
Married	361 (36.3%)	193 (29.7%)	554 (33.7%)		
Divorced/separated/widowed	89 (8.9%)	65 (10%)	154 (9.3%)		
Type of health insurance				2.586	.460
Employee health insurance	339 (34.1%)	215 (33.1%)	554 (33.7%)		
Residents health insurance	275 (27.6%)	203 (31.2%)	478 (29.1%)		
Self-pay	248 (24.9%)	150 (23.1%)	398 (24.2%)		
Others	133 (13.4%)	82 (12.6%)	215 (13.0%)		
Educational level				5.144	.076
Primary school or illiterate	152 (15.3%)	86 (13.2%)	238 (14.5%)		
Middle school	608 (61.1%)	433 (66.6%)	1041 (63.3%)		
College or university or above	235 (23.6%)	131 (20.2%)	366 (22.2%)		
Clinical data					
Comorbid physical diseases				.002	.966
Yes	671 (67.4%)	439 (67.5%)	1110 (67.5%)		
No	324 (32.6%)	211 (32.5%)	535 (32.5%)		
Risky behavior				.009	.924
Yes	607 (61.0%)	395 (60.8%)	1002 (60.9%)		
No	388 (39.0%)	255 (39.2%)	643 (39.1%)		
Total number of episodes				21.109	<.001
1 time	135 (13.6%)	59 (9.1%)	194 (11.8%)		
2–4times	455 (45.7%)	255 (39.2%)	710 (43.2%)		
≥5 times	405 (40.7%)	336 (51.7%)	741 (45.0%)		
Frequency of hospitalization				32.210	<.001
1 time	342 (34.4%)	165 (25.4%)	507 (30.8%)		
2–4times	444 (44.6%)	272 (41.8%)	716 (43.5%)		
≥5 times	209 (21.0%)	213 (32.8%)	422 (25.7%)		
Length of hospital stay (days ± SD)*	44.68 ± 69.87	66.08 ± 85.30	53.13 ± 77.03	-5.331	<.001
General symptoms*	1.95 ± 0.68	1.92 ± 0.70	1.94 ± 0.69	1.010	.313
Hospital admission				2.000	.157
Voluntary hospitalization	196 (19.7%)	110 (16.9%)	306 (18.6%)		
Involuntary hospitalization	799 (80.3%)	540 (83.1%)	1339 (81.4%)		
Insight				1.322	.516
Poor	209 (21.0%)	152 (23.4%)	361 (21.9%)		
Partial	635 (63.8%)	404 (62.1%)	1039 (63.2%)		
Intact	151 (15.2%)	94 (14.5%)	245 (14.9%)		
Received mECT				30.930	<.001
Yes	180 (18.1%)	194 (29.8%)	374 (22.7%)		
No	815 (81.9%)	456 (70.2%)	1271 (77.3%)		

mECT = modified Electroconvulsive Therapy.

* Expressed as mean (standard deviation); the unadjusted *P* value was determined using the *t* test.

**Table 2:** Logistic analysis of demographic and clinical factors in schizophrenia patients receiving combined antipsychotic polypharmacy (N = 1645).

	uOR	95% CI	*P* value	aOR	95% CI	*P* value
Age	0.996	0.989, 1.002	.168	.990	0.980, 1.000	.243
Marital status (Reference: Unmarried)						
Married	0.743	0.598, 0.924	.008	0.795	0.605	.100
					1.045	
Divorced/separated/widowed	1.015	0.719, 1.434	.931	1.049	0.704	.816
					1.561	
Type of health insurance (Reference: Employee Health Insurance)						
Residents health insurance	1.164	0.907, 1.493	.232	1.087	0.822	.558
					1.437	
Self-pay	0.954	0.732, 1.243	.726	1.006	0.744	.970
					1.360	
Others	0.972	0.703, 1.344	.864	0.885	0.618	.504
					1.267	
Educational level (Reference: primary school or lower)						
Middle school	1.259	0.940, 1.685	.122	1.081	0.791, 1.478	.623
College or university or above	0.985	0.701, 1.384	.932	0.880	0.612, 1.266	.491
Comorbid physical diseases (Reference: No)						
Yes	1.005	0.813, 1.241	.996	1.003	0.800, 1.256	.982
Risky behavior (Reference: No)						
Yes	0.990	0.809, 1.212	.924	0.877	0.689, 1.117	.289
Total number of episodes (Reference: 1 time)						
2–4 times	1.282	0.911, 1.806	.154	1.159	0.790, 1.700	.451
≥5times	1.898	1.353, 2.663	<.001	1.380	0.906, 2.101	.134
Total hospitalization frequency (Reference: 1 time)						
2–4 times	1.270	0.999, 1.613	.005	1.152	0.878, 1.510	.307
≥5times	2.112	1.618, 2.757	<.001	1.786	1.253, 2.545	**.001**
Length of hospital stay	1.004	1.002, 1.006	<.001	1.003	1.002, 1.005	**<.001**
General symptoms	0.928	0.804, 1.072	.308	0.901	0.751, 1.081	.260
Hospital admission (Reference: voluntary hospitalization)						
Involuntary hospitalization	1.204	0.931, 1.558	.158	1.179	0.866, 1.605	.295
Insight (Reference: poor insight)						
Partial insight	0.875	0.686, 1.116	.281	0.926	0.708, 1.211	.572
Intact insight	0.856	0.614, 1.193	.358	0.875	0.575, 1.331	.532
Received mECT (Reference: No)	1.926	1.526, 2.432	<.001	1.922	1.502, 2.459	**<.001**

mECT = modified Electroconvulsive Therapy.

Bold indicate statistically significant values (*P* < .05) after data analysis.

## 4. Discussion

To our knowledge, this study is the first to utilize a hospital-based sample to investigate the usage of antipsychotic medications among schizophrenia patients enrolled in China’s 686 Program. Our sample revealed that the majority of patients were treated with SGAs (98.8%). Additionally, 39.5% received polypharmacy with multiple antipsychotic medications, 14.8% were prescribed antidepressants, 20.4% used mood stabilizers, 33.6% were on sedative-hypnotic drugs, and 26.9% took anticholinergic medications. Multivariate analysis revealed that a history of multiple hospitalizations, longer hospital stays, and undergoing mECT during hospitalization were predictors of APP treatment.

Within China’s 686 Program, it was noted that SGAs were predominantly used in treating schizophrenia patients (98.8%), with FGAs being less common (8.3%). This mirrors trends observed in Japan, where SGAs have progressively replaced FGAs over the past 2 decades.^[28]^ Studies suggest SGAs offer superior efficacy and fewer side effects compared to FGAs, resulting in fewer admissions and shorter hospital stays. Furthermore, SGAs are linked to improved medication adherence and lower mortality rates among schizophrenia patients.^[29–31]^ Thus, clinicians may exhibit a more favorable attitude toward SGAs in their prescribing practices.

This study reveals the prevalent use of risperidone (40.8%), olanzapine (30.2%), clozapine (24.6%), and amisulpride (15.4%) as primary antipsychotic medications. While these findings align with those of similar studies in other Asian countries, the notable distinction lies in the utilization of clozapine. For instance, a survey in Korea revealed that the widely used antipsychotic medications among discharged schizophrenia patients were olanzapine, risperidone, and quetiapine.^[32]^ Compared to previous studies of outpatient antipsychotic prescription patterns in China, the main difference also lies in the use of clozapine. Previous studies found that Risperidone was the most frequently prescribed medication, followed by amisulpride and olanzapine.^[33]^ Notably, clozapine’s restricted usage is attributed to severe side effects, including agranulocytosis and weight gain. Its varied usage rates across nations, such as 4.8% in the United States, 0.2% in Japan, and 33.2% in South Africa, contrast with China’s higher rate (24.6%).^[34–38]^ This study also found that the use of clozapine in China corresponds to the proportion of treatment-resistant schizophrenia among the total population of patients with the disorder. Approximately one-third of schizophrenia patients eventually develop into a treatment-resistant, intractable form.^[39]^ Encouragingly, China has witnessed a declining trend in clozapine usage, from 39.7% in 2002 to 26.4% in 2012.^[22]^ This research indicates that this downward trend is continuing. The observed decline in the use of clozapine may be attributed to efforts by clinicians in China to strictly adhere to treatment guidelines and the widespread availability of other SGAs. Future improvements in clozapine utilization could arise from enhanced monitoring protocols, education for clinical practitioners, lifestyle interventions, preemptive management of side effects, and side effect assessment scales.

Treatment guidelines establish AMT as the primary approach for managing schizophrenia, with the consideration of APP, including clozapine, only after exhausting single-agent trials.^[40–42]^ Despite its designation as a last resort, APP is prevalent in clinical practice, driven by factors like primary medication inefficacy, rapid response needs, clozapine intolerance, tapering, side effect management, and comorbidity.^[43]^The elevated usage of APP within our study population represents a significant deviation from existing treatment guidelines. This inclination may stem from the observed challenges with clozapine resistance and the limited efficacy of monotherapy. Our analysis reveals that the predominant antipsychotic combination comprises clozapine and risperidone, indicative of a strategic response to treatment-resistant cases. Furthermore, our multivariate analysis underscores the predictive value of APP treatment in identifying resistance patterns within the disease spectrum. However, research indicates that APP may escalate adverse reactions, costs, hospitalizations, and mortality rates compared to AMT.^[44]^ The use of APP remains highly controversial in clinical practice. The global prevalence of APP is 19.6%, with 32% in Asia, 23% in Europe, 16% in North America, and 16.4% in Oceania.^[45]^ Our sample shows an APP usage rate of 39.5%, which is higher than in many other countries. Moreover, it was found that the rate of APP use in China appears to be increasing over time, from 26.1% in 2002 to 34.2% in 2012,^[46,47]^ and this upward trend continues. The high rate of APP use may reflect the proactive treatment efforts of psychiatrists in China when faced with more severe or treatment-resistant cases of schizophrenia. Even after the resolution of acute psychosis or treatment-resistant episodes through APP, many clinicians are reluctant to reduce antipsychotic medications, leading to many patients continuing with APP.^[48]^ Notably, this study found that patients on APP used clozapine more frequently compared to those on AMT, reflecting to some extent that psychiatrists in China are following the guidelines by initiating APP after unsuccessful trials of clozapine monotherapy. To tackle the divergent findings regarding app benefits and drawbacks, it’s crucial to emphasize tapering’s role in reducing multiple drug-related side effects to psychiatrists. Future research should prioritize randomized controlled trials to determine the safest, most effective antipsychotic combinations, while also identifying patient characteristics that can optimize these combinations for improved outcomes.

Antipsychotic medications are primary treatments for schizophrenia, supported by robust evidence. Yet, they often fall short in addressing all symptoms and functional impairments alone. Integrating alternative psychiatric medications, such as antidepressants, mood stabilizers, and sedative-hypnotics, diverges from typical guidelines but is common in clinical practice.^[49,50]^ This research found that, in addition to antipsychotics, 14.8% of patients received antidepressants, 20.4% mood stabilizers, and 33.6% sedative-hypnotics. Antidepressants target depressive or negative symptoms, mood stabilizers address excitation, agitation, or aggression, and sedative-hypnotics treat comorbid anxiety or insomnia.^[20]^ However, evidence supporting these approaches is limited, lacking sufficient data on side effects and long-term safety for routine clinical use.^[51]^ Some scholars suggest considering adjunctive therapies only if symptoms persist after antipsychotic treatment.^[52]^ Monitoring and weighing therapeutic benefits against side effects are crucial when using non-antipsychotic psychiatric medications in schizophrenia patients.

It is noteworthy that this research revealed that 26.9% of patients used anticholinergic medications. While this is lower than previous surveys conducted in Asia (66.3% in 2001, 52.8% in 2004, and 54.6% in 2009),^[53]^ it is still higher than in some Western countries (11.7% in 1996, 5.7% in 2012).^[54]^ The high frequency of anticholinergic prescriptions can be explained by several factors. Firstly, the rate of APP use in this study is significantly higher than in Western countries. Research suggests that one of the clinical drawbacks of APP is the higher severity of side effects,^[55]^ which may exacerbate extrapyramidal side effects. Another reason may be that Asian patients have a lower threshold for treatment and adverse reactions to antipsychotic medications compared to populations in Western countries,^[56]^ necessitating the greater use of anticholinergic medications to treat and prevent extrapyramidal side effects. Given the increased risk of sedation and cognitive impairment associated with anticholinergic medications,^[57]^ and the recommendation against their prophylactic or long-term use in treatment guidelines,^[58]^ clinicians should exercise caution when prescribing them.

In our multivariate analysis, we found that patients with a history of multiple hospitalizations and longer hospital stays were more likely to receive APP, consistent with the findings of Ching-Hua Lin et al^[59]^ and Patrichi et al.^[21]^ Additionally, patients who underwent mECT during hospitalization were also more likely to be treated with APP. Multiple hospitalizations often indicate severe psychopathology and recurrent issues resistant to improvement, suggesting treatment resistance.^[60]^ Longer hospital stays may signify incomplete treatment responses and disease intractability.^[61]^ For treatment-resistant schizophrenia, combining antipsychotic medications with ECT has been considered, showing higher rates of clinical improvement compared to antipsychotic medications alone.^[62,63]^ Thus, patients treated with ECT may also exhibit treatment resistance. It is plausible that patients treated with APP experience less therapeutic improvement and have more severe and complex diseases compared to those receiving AMT.

### 4.1. Limitations

This study has several limitations. First, the study was retrospective and lacked complete clinical records, such as data on daily drug doses, information on treatment resistance and side effects was not obtained, which hindered the assessment of APP suitability. However, the large sample allows for multivariate statistics. In addition, this was a single center experience and most patients were from the same province. The results of the current study need to be confirmed by a prospective multicenter study.

## 5. Conclusion

APP is common among schizophrenia patients enrolled in Guangdong Province, China’s 686 Program. The findings of this study suggest that there are interactions between the sociodemographic and clinical characteristics of patients associated with APP in the 686 Program. Given the heterogeneous findings in contemporary research regarding the advantages and drawbacks of APP, it is imperative to enlighten psychiatrists about the significance of appropriate medication tapering to mitigate polypharmacy-related adverse effects. Moreover, additional investigation is warranted to delineate optimal approaches for integrated polypharmacy practices and augmented treatment modalities with other psychiatric medications tailored to this specific patient cohort.

## Author contributions

**Conceptualization:** Xuanyu Xu.

**Data curation:** Junyu Li.

**Formal analysis:** Shujia Zhang.

**Funding acquisition:** Jinghua Su.

**Investigation:** Jinghua Su.

**Methodology:** Yingji Hu.

**Project administration:** Xiaodong Chen.

**Writing – original draft:** Yingji Hu, Xiaodong Chen.

**Writing – review & editing:** Yingji Hu.
